# Timescales of Quartz Crystallization and the Longevity of the Bishop Giant Magma Body

**DOI:** 10.1371/journal.pone.0037492

**Published:** 2012-05-30

**Authors:** Guilherme A. R. Gualda, Ayla S. Pamukcu, Mark S. Ghiorso, Alfred T. Anderson, Stephen R. Sutton, Mark L. Rivers

**Affiliations:** 1 Vanderbilt University, Earth & Environmental Sciences, Nashville, Tennessee, United States of America; 2 OFM Research - West, Seattle, Washington, United States of America; 3 The University of Chicago, Geophysical Sciences, Chicago, Illinois, United States of America; 4 The University of Chicago, Center for Advanced Radiation Sources, Argonne, Illinois, United States of America; University of Leeds, United Kingdom

## Abstract

Supereruptions violently transfer huge amounts (100 s–1000 s km^3^) of magma to the surface in a matter of days and testify to the existence of giant pools of magma at depth. The longevity of these giant magma bodies is of significant scientific and societal interest. Radiometric data on whole rocks, glasses, feldspar and zircon crystals have been used to suggest that the Bishop Tuff giant magma body, which erupted ∼760,000 years ago and created the Long Valley caldera (California), was long-lived (>100,000 years) and evolved rather slowly. In this work, we present four lines of evidence to constrain the timescales of crystallization of the Bishop magma body: (1) quartz residence times based on diffusional relaxation of Ti profiles, (2) quartz residence times based on the kinetics of faceting of melt inclusions, (3) quartz and feldspar crystallization times derived using quartz+feldspar crystal size distributions, and (4) timescales of cooling and crystallization based on thermodynamic and heat flow modeling. All of our estimates suggest quartz crystallization on timescales of <10,000 years, more typically within 500–3,000 years before eruption. We conclude that large-volume, crystal-poor magma bodies are ephemeral features that, once established, evolve on millennial timescales. We also suggest that zircon crystals, rather than recording the timescales of crystallization of a large pool of crystal-poor magma, record the extended periods of time necessary for maturation of the crust and establishment of these giant magma bodies.

## Introduction

Supereruptions [1] provide compelling evidence that giant bodies of low-density, crystal-poor magma – larger than currently known magma bodies (see [2]) – existed just a few kilometers below the surface. The generation of such large pools of magma and their eruption are fascinating phenomena from a scientific standpoint, but also constitute a major threat to humanity [1], [3]. Our knowledge of the processes and consequences related to these events is limited, largely because the last known supereruption on Earth occurred ca. 26,000 years ago [1]. The 1815 eruption of Tambora – the largest known historic eruption – led to the “Year without a summer” in North America and Europe in 1816 [4]. Yet, the Bishop supereruption, which took place ∼760,000 years ago and created the Long Valley caldera in California [5]–[7], ejected at least an order of magnitude more magma, demonstrating that these giant eruptions can not only cause widespread devastation at the local scale, but can also have a significant worldwide impact, particularly on climate [1], [3].

The longevity of giant magma bodies has generated continued interest [8], [9]; the Bishop Tuff, in particular, has been the focus of numerous studies using age-dating (for a recent review, see [10]). One of the continuing puzzles is that the timescales inferred are not always consistent with each other [10], [11]. While ion probe U-Pb zircon ages suggest zircon crystallization over >100 ka [10], whole-grain TIMS U-Pb zircon ages combined with Ar-Ar sanidine eruption ages suggest millennial timescales [11]; however, the latter results critically depend on the intercomparability of U-Pb and Ar-Ar ages, a point of significant contention [12].

In discussion here is also the potential geological significance of the various results. What are the timescales of assembly of a giant magma body? What are the timescales of crystallization of such a magma body? The challenge is that the timescales of interest are largely inaccessible to geochronology for deposits as old as the youngest supereruptions. These timescales are accessible, however, using kinetic markers of magmatic processes (i.e. geospeedometers, see [13]). We present here direct evidence of the timescales of quartz crystallization in the Bishop Tuff magma body, and we compare these timescales to those expected from simple thermodynamic and heat flow calculations. Our results shed light onto the questions above and have significant implications for the timescales over which eruptible crystal-poor magma resides in the upper crust.

## Materials and Methods

### Samples

Our sample set includes 6 pumice clasts from the Chalfant Quarry in the southeastern portion of the Bishop Tuff [14], [15], and 5 pumice clasts from the Aeolian Buttes in the northern portion of the deposit [16]. In the stratigraphic classification scheme of Wilson & Hildreth [7], our samples derive from fall unit F7 (2 pumice clasts), fall unit F8 (1), ash-flow unit Ig2Ea (3), and ash-flow unit Ig2NWb (5).

Studied samples encompass much of the spectrum of pumice density, porosity, crystallinity and textural variations observed in the Bishop Tuff as a whole [14]. Crystal size distributions for the samples from Chalfant were previously determined by us [14], [15], and we discuss recent results on crystal size distributions for samples of the Aeolian Buttes [16], [17]. Quartz zoning data presented here are exclusively for samples from Chalfant. Melt inclusion shape information derives from observations partly reported by Anderson et al. [18].

### Analytical methods

Samples were studied using a combination of (for details, see [19]):

1. Documentation of sizes and shapes of whole quartz crystals and their melt inclusions; we place whole crystals in refractive index oil (so as to emphasize the inclusions), and we make observations under a petrographic microscope; over the years, we have inspected hundreds of crystals, each one containing tens of inclusions, so we have made qualitative observations on thousands of inclusions; we have only characterized in detail a small number of inclusions;
2. Cathodoluminescence (CL) imaging of individual quartz crystals (∼100 in total) by electron microprobe at The University of Chicago, using methods similar to those of Peppard et al. [20];
3. Trace-element analysis at low spatial resolution (∼25 µm) by laser ablation mass spectrometry (LA-ICPMS);
4. Trace-element analysis along traverses and 2D maps (4 crystals studied) at high spatial resolution (ca. 2–10 µm spacing) using synchrotron x-ray microfluorescence (x-ray microprobe; see [21]) at the GeoSoilEnviroCARS insertion device beamline at the Advanced Photon Source (Argonne National Lab); the x-ray microprobe uses a highly collimated, synchrotron-based x-ray beam to generate characteristic x-ray spectra with very low background, which yields 2σ uncertainty close to 2.5% for ca. 50 ppm Ti in quartz, using a 5 µm spot [21];
5. X-ray tomography of pumice chips at various resolutions (2.5 to 15 µm per voxel), performed at the GeoSoilEnviroCARS bending magnet beamline, using methods described elsewhere [15], [16].

### MELTS calculations

Our ongoing effort to use MELTS to model the evolution of silicic systems has shown that the current calibration of MELTS [22] fails to correctly predict the quartz+feldspar saturation surface as a function of pressure. We recently developed a new calibration of MELTS, rhyolite-MELTS, optimized for fluid-bearing rhyolitic magmas [23]. Our calibration takes advantage of the overwhelming evidence for simultaneous saturation of quartz, sanidine, and plagioclase in early-erupted Bishop pumice, and the well-constrained pressure and fluid phase composition and abundance from melt inclusions [24], [25]. Our tests indicate that the corrections lead to much improved results not only for Bishop magma, but also for other silicic systems [23].

We use rhyolite-MELTS to constrain the crystallization paths, and, in particular, the heat of cooling and crystallization for compositions relevant for the Bishop Tuff. We use early- and late-erupted bulk pumice (from [6]) and melt inclusion (from [18]) compositions. Calculations using the H_2_O-CO_2_ solubility model of Papale et al. [26] show that both early- and late-erupted melt inclusion compositions yield fluid-saturation under the conditions inferred [24], [25]. Unfortunately, we are currently unable to model a H_2_O-CO_2_ fluid phase; however, the solubility of CO_2_ in melts is so low [27] that the phase relations are virtually unchanged [28], [29]. Accordingly, in our simulations, we add H_2_O to the initial compositions until rhyolite-MELTS calculates fluid saturation; the effect is that the activity of water in the melt is buffered throughout crystallization, as would be the case in the presence of a H_2_O-CO_2_ fluid (for a more extended discussion, see [23]).

Crystallization pressure is determined using rhyolite-MELTS as the pressure at which melt inclusion compositions show simultaneous crystallization of quartz+sanidine+plagioclase under fluid-saturated conditions (see [23]).

## Results

### Timescales of quartz crystallization

To assess the timescales of quartz crystallization and their implications for the longevity of giant rhyolitic magma bodies, we analyze three lines of evidence: (1) quartz residence times based on the diffusional relaxation of Ti zoning profiles; (2) melt inclusion faceting timescales, over which initially round melt inclusions attain partly faceted shapes; (3) quartz+feldspar crystallization times as recorded in crystal size distributions. We discuss the theory behind residence time and melt inclusion faceting timescale calculation in some detail, and highlight the connection of these results with those obtained using crystal size distributions, which are detailed elsewhere [16], [17].

#### Diffusional relaxation times

**Figure 1:**
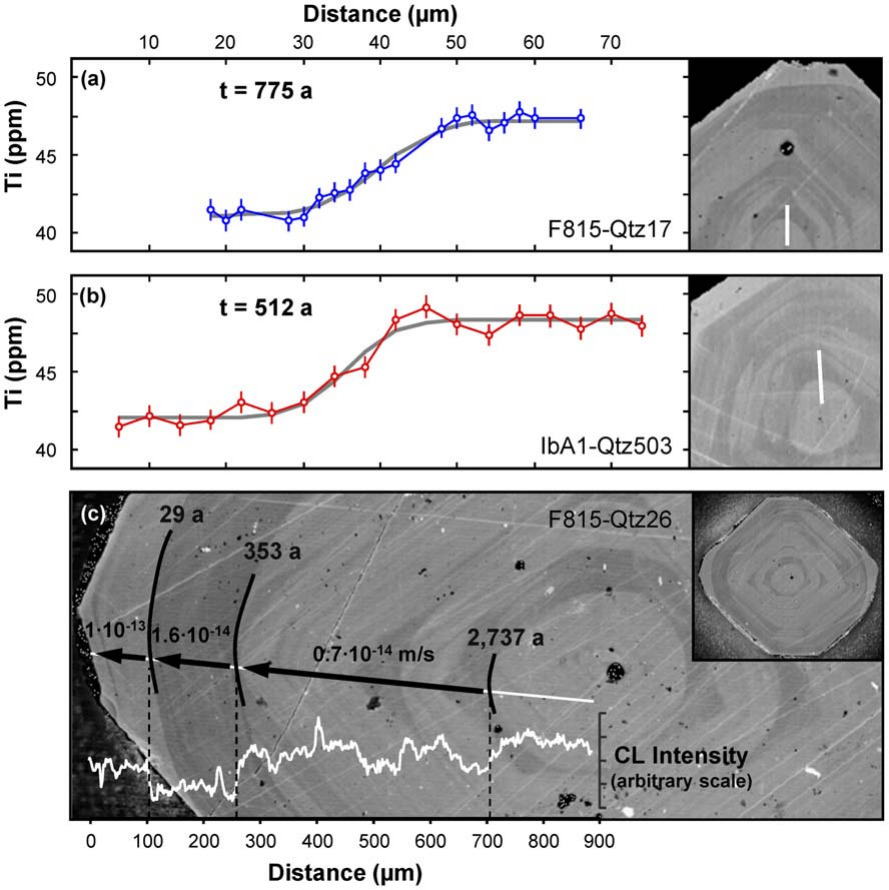
Diffusional relaxation of Ti in quartz. (a–b) X-ray profiles and cathodoluminescence (CL) images of select quartz crystals with bright-CL, high-Ti cores; residence times and growth rates derived from Ti traverses (white lines) are presented. Analytical points shown by open symbols, including analytical uncertainties (bars). Best fit curve (Equation 1) is shown in gray, calculated so as to minimize the sum of the squares of the difference between calculated and observed values. Calculated residence times are also shown. (c) CL image detailing core-rim zoning of a large quartz crystal; image of whole crystal shown in inset. White line corresponds to the location of CL traverse displayed in the bottom, with contacts between different zones indicated in black. Residence times in years and derived growth rates indicated by numbers on top of arrows. Notice that innermost contact has residence time close to 3,000 years. Calculated growth rates for two interior zones are close to 10^−14^ m/s, while growth rate for rim is ∼10^−13^ m/s.

Quartz crystals are characterized by a volumetrically predominant low-Ti (∼40 ppm Ti) interior portion (Fig. 1). A small fraction (<10%) of crystals also include a relatively high-Ti (∼50 ppm Ti) central core (50–100 µm in diameter); in the absence of such a core, the interior portion extends to the center of the observed crystal section. Finally, a variable proportion of crystals show a 50–100 µm thick rim with high Ti (∼50 ppm Ti), which corresponds to the bright cathodoluminescence (CL) rims first recognized by Peppard et al. [20]. Similar rims are observed on sanidine crystals. The origin of such features is contentious, and to what extent they record replenishment of the magma body [30], [31], sinking of phenocrysts [18], or decompression of the system [19], [32] is yet to be established. Nonetheless, quartz crystallization timescales can be inferred using diffusional relaxation times of Ti zoning in quartz from early-erupted Bishop pumice (Fig. 1).

*Residence time calculation.* The sharpness of contacts between chemically distinct zones in crystals can constrain the residence times of these internal contacts, and the durations and rates of crystal growth. The rationale is that once established, concentration gradients tend to relax by diffusion (e.g. [33]); diffusivities are such that relaxation is significant while at magmatic conditions, but becomes negligible after eruption, such that timescales and rates relevant to magmatic processes can be retrieved. In the present case, the problem is particularly simple because quartz has uniform major element composition, and there is no need to consider potential effects of composition on the partition or diffusion coefficients.

Diffusion of Ti in quartz is particularly useful for the problem of interest here, as (1) diffusion rates have been determined experimentally (i.e. D_Ti_^Qtz^ = 8.05×10^−22^ m^2^ s^−1^ at 750°C; see [34]), and are adequate to constrain timescales in question; and (2) Ti contents in quartz, in concentrations of 10 s of ppm, while not trivial to measure, can be measured with sufficient precision at the micrometer scale using the x-ray microprobe. Because large variations in Ti concentration in quartz correlate well with large changes in CL intensity [19], [35], CL images and profiles can, in favorable instances, be used as a proxy for Ti variations. We use both Ti profiles obtained using the x-ray microprobe and CL intensity profiles derived from CL images, which allows us to study a larger number of crystals than would be possible with Ti profiles alone. Profiles were selected so as to be approximately orthogonal to the contact between different zones; departures from orthogonality are small and their effect on calculated times can be effectively neglected.

We use a 1D diffusion model to determine the time interval during which two zones of distinct compositions within a crystal were in contact with each other. We call this the contact residence time. We assume constant but different initial compositions in the two zones of the crystal at time t = 0. The predicted composition c(x) as a function of position x along the profile is described by [36]:
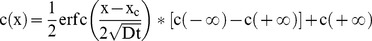
(1)where erfc is the complementary error function, x_c_ is the position of the initial contact between the two zones (e.g. the position of the initial jump in composition), D is the diffusion coefficient, t is time, and 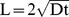 is the characteristic diffusion length scale. The predicted composition described by Equation 1 is fit to the observations by iteratively adjusting x_c_, c(−∞), c(+∞), and L so as to minimize the sum of the squares of the differences (SSD) between observed and predicted composition. Using the known diffusivity D, we infer the diffusion time from the best-fit value of L. This fitting procedure makes it possible to recover estimates of L that are smaller than the spacing of the profile. We test whether L is appropriately resolved by varying its value about the best-fit value; when the sum of the square of the differences between observed and fitted curves increases with both larger and smaller values of L, we conclude that the diffusion time is resolved, even if L is smaller than the sampling interval; alternatively, this test may show that reduction of the diffusion distance leads to no change in the SSD, and the obtained value is simply a maximum estimate.

*Error propagation.* It is of great interest to assess the errors associated with the calculated residence times. In order to derive an expression for the uncertainties associated with the residence time t, we re-write the equation 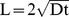 as an explicit function of temperature (T) by using the Arrhenius relation D = D_o_ exp [−E/RT]):
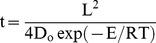
(2)where D_o_ is the pre-exponential factor and E is the activation energy for diffusion, both of which have been determined experimentally [34]. Error propagation shows that:
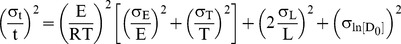
(3)Uncertainties on E and ln(D_0_) come directly from experiments (i.e. E = 273±12 kJ mol^−1^, σ_E_/E = 4.4%, log[D_0_] = −7.154±0.525, σ_ln[D0]_ = 1.2; see [34]), while the other parameters have to be estimated. Best estimates for the crystallization temperature of the Bishop magma are in the range 720–780°C [6], [37]. We choose a temperature of 750°C, and an uncertainty of 15°C (1σ), such that the resulting 95% confidence interval matches the estimated T range. This calculation yields σ_T_/T = 1.5%, much smaller than the other contributions. Uncertainties on L derive from the fitting procedure. For Ti profiles, a Monte Carlo procedure was used to estimate the effect of the uncertainty at each point on L, which results in estimated errors in L of ca. 10–50%. Because the focus is not on the individual dates, but rather on the best estimate for the age of a single event recorded by several profiles, the standard deviation of the mean is a more appropriate measure of uncertainty; the standard error is typically between 1 and 4%.

For the conditions of interest, the factor (E/RT)^2^ is ca. 1000, such that even though the uncertainty on L is much larger than those on E, T, and ln[D_0_], all terms are potentially important. Even if σ_T_ is chosen to be 2 or 3 times larger than our choice, its effect on the final uncertainty is relatively minor, and the contributions of uncertainties on E, ln[D_0_] and L dominate. For (σ_E_/E) = 4.4%, (σ_T_/T) = 1.5%, (σ_L_/L) = 50%, and σ_ln[D0]_ = 1.2, the uncertainty on t becomes (σ_t_/t) = 215%; for (σ_L_/L) = 5%, (σ_t_/t) = 190%. Even if (σ_L_/L) could be improved, (σ_E_/E) and σ_ln[D0]_ are such that the total uncertainty would not be better than ∼190%, showing that our estimates are of appropriate precision for evaluating the parameters of interest. Furthermore, even though these uncertainties are large, our knowledge of the timescales being investigated is minimal, and the estimates remain meaningful. Importantly, none of the conclusions drawn are affected by the particular choice of parameters used.

*Relaxation times and growth rates.* We focus here on core-interior contact residence times, while results for interior-rim contacts, which have been previously studied [31], [38], will be detailed elsewhere. These core-interior contact residence times mark – to a good approximation – the onset of crystallization of individual quartz crystals; this is true regardless of how complex the crystallization history may have been (e.g. affected by resorption events), as long as magmatic temperatures were sustained; our calculations are robust provided the boundary conditions used are valid.

Core-interior contact residence times were estimated primarily using Ti profiles of a selected number of crystals, but also using CL images of other crystals. The most important results are shown in Fig. 1 and Table 1. Typical values for the core-interior residence times are 500–1,000 years, for crystals ca. 1 mm wide (Fig. 1a–b). The largest calculated time is that shown in Fig. 1c, i.e. ∼2,700 years, for a crystal ca. 2 mm wide. Our analysis suggests that uncertainties are close to 200%, leading to a 95% confidence interval with upper bound close to 13,000 years for the maximum time we estimate, and more typically <5,000 years.

**Table 1:** Residence times of internal contacts, widths, growth times and growth rates of selected quartz crystals (two of which shown in Fig. 1), as derived from Ti traverses.

CRYSTAL		CORE-INTERIOR CONTACT			
*Label*	*Fig. 1*	*Residence time* a	*Interior width*	*Interior growth time* b	*Interior growth rate* c
		*(years)*	*(µm)*	*(years)*	*(10^−14^ m/s)*
F815-Qtz-17	(a)	775 [3747]	400	765	1.5 [0.3]
IbA1-Qtz-503	(b)	923 [4461]	334	922	1.0 [0.2]
IbA1-Qtz-518		512 [2477]	260	506	1.3 [0.3]

a Quantity in brackets is the maximum residence time: t+2σ_t_.

b Interior growth time excludes the time estimated for the residence time for rim-interior contacts in crystals with bright-CL, high-Ti rims (Gualda et al., unpublished data); these times are short enough that interior growth rates would be unaltered even if they were neglected.

c Quantity in brackets is the minimum growth rate obtained using t+2σ_t_ as time.

Our core-interior contact residence times are 1–2 orders of magnitude larger than – and thus consistent with – the quartz rim-interior residence times of Wark et al. [31]. They are, however, inconsistent with the results of Morgan & Blake [38], who calculate sanidine rim-interior residence times of ∼200 ka; more recent data on quartz and sanidine zoning [19], [31] shows zoning profiles that are much sharper than those of Anderson et al. [18] used by Morgan & Blake [38], putting into question their calculated timescales.

Importantly, because the growth distance is known for the interior regions, the contact residence times can be used to constrain average growth rates, an important parameter for which estimates are entirely lacking for quartz in large-volume silicic systems like the Bishop Tuff. The agreement between the interior growth rates is remarkable, with all estimates close to 10^−14^ m/s (Fig. 1; Table 1). These values fall within the constraints placed by the analysis of Anderson et al. [18], and are also in good agreement with growth rates for plagioclase phenocrysts in much smaller dacitic systems [39]. The agreement between these growth rates suggests that the crystallization process was rather monotonic, with no sharp discontinuities due to recharging and resorption events – which is consistent with the evidence for phenocryst homogeneity within individual pumice clasts of the Bishop Tuff [6].

#### Melt inclusion faceting times

**Figure 2:**
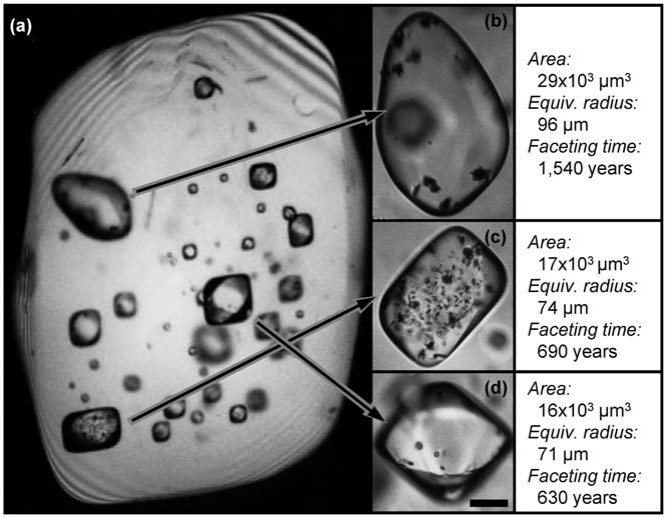
Examples of melt (glass) inclusions in quartz at different stages of faceting. (a) Quartz crystal in refractive index oil (cross-polarized light) showing several melt inclusions. (b–d) Detailed views of the three largest inclusions; scale bar is 50 µm and applies to all 3 images; area, radius (of a circle with same area), and faceting time are indicated for each inclusion. Note that (b) is non-faceted, (c) is partly faceted, and (d) is faceted. That only (d) is faceted suggests that crystal residence times are <1,500 years. Images (a–d) are from Anderson et al. [18], reproduced with permission.

**Figure 3:**
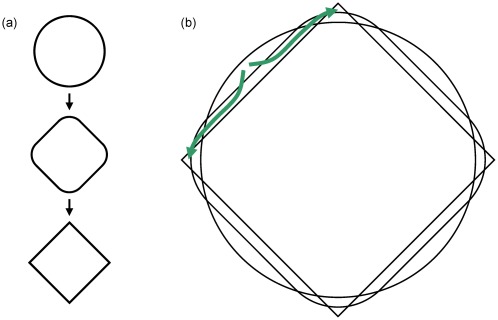
Shape evolution due to melt inclusion faceting. (a) Shape change of a melt inclusion inside a host crystal as a function of time due to faceting; initial inclusion is spherical, but with time gets transformed into a polyhedron with rounded edges; with sufficient time, inclusion may become a perfect anticrystal. (b) Evolution of shapes emphasizing the role of diffusion (green arrows) in transporting material to achieve faceting.

Quartz crystals from the Bishop Tuff are rich in melt inclusions (now glass), which have been extensively studied by Anderson and co-workers (e.g. [18], [24], [25]), among others (e.g. [30]). One striking observation is that melt inclusions show a variety of shapes, from round to faceted, sometimes within the same crystal (Fig. 2). For instance, Anderson et al. [18] observed that “*Several late-erupted quartz phenocrysts have clear, faceted inclusions located far from crystal rims and round, brown inclusions near the rims of the same crystals*”. Their observations are suggestive of shape maturation (Fig. 3), during which initially round melt inclusions gradually become faceted (i.e. assume negative crystal shapes) by dissolution and reprecipitation [40]–[42].

Importantly, many of the larger central melt inclusions are round [18], indicating that only smaller melt inclusions became faceted. This coexistence of round and faceted melt inclusions reveals that the timescale of melt inclusion faceting is similar to the residence time of quartz hosts. Faceting depends on diffusion [42], particularly of Si atoms in the case of quartz, such that significant faceting occurs only at relatively high (i.e. magmatic) temperatures. Hence, determination of melt inclusion faceting times ultimately provides an independent assessment of the longevity of the Bishop magma.

*Kinetics of melt inclusion faceting. Melt* inclusion faceting is the result of surface free energy minimization. Faceting can be spontaneous if the increase in total surface area – due to conversion from spherical to polyhedral shape – is more than compensated by the accompanying reduction in specific surface energy due to replacement of curved surfaces by flat surfaces [43]. The kinetics of melt inclusion faceting has not been treated in detail, and a full treatment is beyond the scope of this presentation. What we seek is a simplified treatment that can reveal an order of magnitude assessment of the time required for melt inclusion faceting and, consequently, of the residence times for quartz crystals in the Bishop magma.

We note that the problem involves lateral diffusion of material from the spherical cap that is gradually dissolved into the corner region that progressively forms by reprecipitation (Fig. 3b). We use the volume of the spherical cap that sticks out of the flat surface of the polyhedron of same volume as an estimate of the total volume that needs to be diffused; this is a limiting case that yields maximum faceting time, as the equilibrium shape may be a rounded – rather than a perfect – polyhedron [44]. We then use Fick's first law in combination with the Thomson-Freundlich equation to derive an approximate kinetic expression – similar to the solution for coarsening of a single particle in a matrix [45].

The volume change ΔV can be calculated as the volume of the 12 spherical caps that stick out of the flat surfaces of a hexagonal bipyramid of side a and height h:
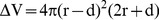
(4)where r is the radius of the sphere and d is the distance between the center of the bipyramid and a flat surface, given by:
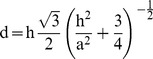
(5)Because we assume equal volumes for the bipyramid and the sphere, we find that:
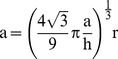
(6)which shows that the volume to be diffused depends only on the size of the original inclusion (r) and the ratio a/h. For quartz, we use a value of 0.909 for a/h.

The flux equation simply states that material transport is by diffusion [45]:
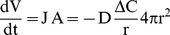
(7)where V is volume, t is time, J is the flux of material per unit area and A is the area of the particle such that JA is the total flux of material through the surface of the particle, D is the diffusion coefficient, and ΔC is the concentration difference that drives the process – in units of mass or mol fraction.

The Thomson-Freundlich equation [46] describes the quantity ΔC as a function of particle size:
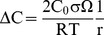
(8)where C_0_ is the solubility of a particle of infinite radius, σ is the surface energy, Ω is the molar volume of the phase of interest, R is the ideal gas constant, and T is temperature.

**Figure 4:**
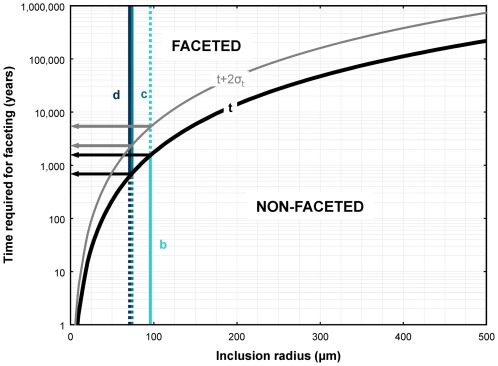
Melt inclusion faceting time. Time required for faceting versus inclusion radius plot for conditions relevant for Bishop magma crystallization. Vertical lines correspond to inclusion sizes estimated from Fig. 2. Using our best estimate of faceting time for the observed inclusions, we constrain the timescale for residence of the host crystals to be between ∼600–1,500 years. Even if faceting is significantly slower (t+2σ_t_ curve), residence times are within the range ∼2,200–5,300 years.

Combining Equations 7 and 8, and assuming that the scaling is appropriate for non-infinitesimal changes, we can calculate the faceting time as:
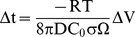
(9)The solution to Equation 9 is plotted in Fig. 4, where we used the somewhat conservative values shown in Table 2.

**Table 2:** Parameters used and estimated uncertainties for the computation of melt inclusion faceting time as a function of inclusion radius (r).

	X	σ_X_	Unit	σ_X_/X	Source
r	100	-	10^−6^ m		
ΔV	4.8 10^−13^	-	m^3^	∼10%^a^	
R	8.31451	-	J/(K·mol)	-	
T	750	15	°C	2%	
C_0_	0.7	∼0.2^a^	.	∼30%^a^	
σ	0.02	0.01	J/m^2^	50%	[*71*]
Ω	23.7	1.0	10^−6^ m^3^/mol	4%	b
H_2_O	3	-	Wt. %		
D_0_	25.8		10^−9^ m^2^/s		[*72*]
ln[D_0_]	−17.5	0.70		4%	[*72*]
E	126.5	8.5	10^+3^ J/mol	7%	[*72*]
D	1.28	1.06	10^−14^ m^2^/s	83%	[*72*]
(E/RT)^2^	132.6	.	.	.	.
Δt	1,242	1,260	a	101%	

a Approximate values.

b Calculated using the CORBA Phase Properties applet (http://ctserver.ofm-research.org/phaseProp.html). Retrieved Nov 12, 2007. Calculations based on data from [73].

*Error propagation.* We use error propagation to assess how sensitive the computations are to uncertainties in the various parameters in Equation 9, which results:
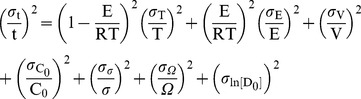
(10)For the conditions of interest (Table 2), (E/RT)^2^ = 138 and (1−E/RT)^2^ = 115, such that the uncertainties in E and T are of much greater importance than what the values of σ_X_/X may suggest. An analysis similar to that carried out above for diffusional relaxation shows that the final error, which is ca. 125%, is dominated by the uncertainties in E and ln[D_0_].

*Faceting times.* Our calculations of faceting times show that melt inclusions with 50, 100, and 175 µm radius would become faceted in as little 200, 1,700, and 10,000 years, respectively (Fig. 4). That the largest central melt inclusions are not faceted suggests that quartz residence times in the Bishop magma were well below 10,000 years.

Specific results for the melt inclusions shown in Fig. 2 are also plotted in Fig. 4. The non-faceted inclusion (Fig. 2b), which has an equivalent radius (i.e. radius of a circle with the same area as the inclusion) close to 100 µm, suggests that it was entrapped less than 1,500 years before eruption. The faceted and partially faceted inclusions (Fig. 2c–d), on the other hand, with equivalent radii ∼70–75 µm, suggest residence times in excess of ∼600 years.

Even if, for sake of argument, we assume that t values are underestimated by 250% (+2σ), our conclusions do not significantly change, as illustrated in Fig. 4. In this case, instead of a residence time of 600–1,500 years, we would infer a residence time of 2,200–5,300 years for the crystal in Fig. 2, and even inclusions with 170 µm radius would become faceted in ca. 10,000 years.

#### Crystal size distributions

**Figure 5:**
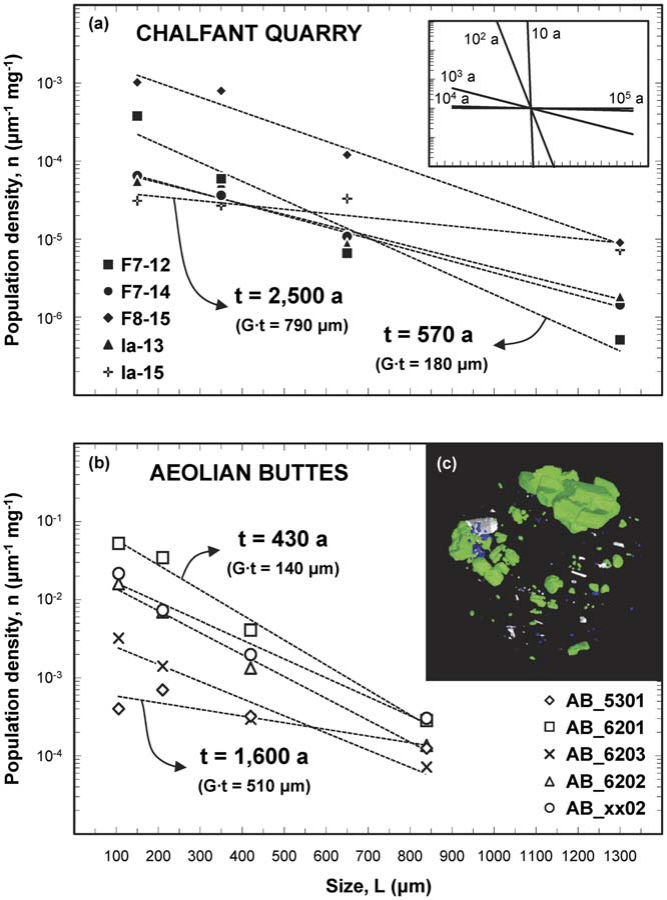
Crystal size distributions. (a) Whole-quartz crystal size distributions for pumice from Chalfant Quarry, obtained by a crushing, sieving and winnowing procedure (data from [14]). (b) Quartz+feldspar crystal size distributions for pumice from Aeolian Buttes, obtained by x-ray tomography (data from [16], [17]). (c) 3D view of pumice chip (sample AB-6203F), showing euhedral quartz and feldspar grains (green), magnetite (blue), and pyroxene±biotite (white); notice the overall trend of decreasing numbers of crystals with increasing size; sample is approximately cylindrical, field of view ∼9 mm (see Movie S1 for animated version). In (a) and (b), only crystals larger than 35 µm are shown. Crystal size distributions are well-approximated by exponential distributions (dashed lines). Using growth rates of ∼10^−14^ m/s (as calculated above), we calculate crystallization times between 430 and 2,500 years, as indicated; only maximum and minimum estimates shown. Inset in (a) shows slopes of distributions with crystallization times of 10–100,000 years (for G = 10^−14^ m/s), demonstrating that crystallization times in excess of 10,000 years would yield effectively horizontal size distributions at the scale used, in contrast with the crystal size distributions obtained by us.

Crystal size distributions in rocks are typically characterized by a monotonic decrease in the number density of crystals with size, with numerous small crystals and few large crystals (see [47] and references therein, among many others). Due to this pronounced decrease in crystal number with increasing size, we use bin sizes that increase by a factor of 2 with increasing size, similar to the practice employed in sedimentology; this yields more robust crystal size distributions, in which the uncertainties in each bin size are similar to each other, rather than quickly increasing with size as would happen with equal bin sizes (for details, see [48]). The crystal size distributions reveal a two-stage growth history for quartz and sanidine [17], with crystals >200 µm recording a simple history of growth under small supersaturation (undercooling) with limited nucleation, characteristic of the pre-eruptive crystallization of a giant magma body with large thermal inertia [14]. Both early- and late-erupted Bishop pumice display such trends (Fig. 5) [14], [16], [17]. As long as growth rates can be inferred, quartz crystal size distributions yield additional information on the timescales of crystallization [47], and also provide clues to the significance of these timescales.

Theoretical and observational considerations [47] provide compelling evidence that the usual exponential trends (linear in semi-log plots of population density versus size) require linear growth rates with time, as inferred above using relaxation times. That being the case, the largest crystals present are the oldest, and knowledge of growth rates and maximum crystal size leads to estimates of crystal growth time. While determination of maximum size is plagued by extremely incomplete sampling of the existing >600 km^3^ of erupted magma, crystal size distributions provide constraints on the abundance of such unlikely large crystals. Our data suggest that crystals up to 2 mm in diameter comprise at least 95% of the total crystal mass, and crystals larger than 3 mm (none found in our samples) are exceedingly rare, corresponding to <0.1% of the crystal mass. The growth rates above imply maximum growth times of less than 5,000 years even for 3 mm crystals, such that >99% of the observed crystallization would take place within this time frame.

Additional evidence for the timescales of quartz crystallization derive from crystal size distributions, given that the slope in semi-log space decreases systematically with time [47], [49]:
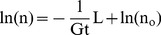
(11)where n is population density (i.e. number of crystals per mass per bin size), L is size, G is crystal growth rate (in length per time) and n_o_ is the intercept at L = 0. We calculate best-fit exponential curves to quartz crystal size distributions for pumice from Chalfant [14] and to quartz+feldspar size distributions for pumice from Aeolian Buttes [16], [17]; using the growth rates calculated above, we infer a duration of the crystallization event between 400 and 2,500 years (Fig. 5) [17].

Our calculations based on crystal size distributions thus suggest residence times for quartz crystals in the millennial scale, in remarkable agreement with our estimates based on diffusional relaxation and melt inclusion faceting, lending confidence to our results. Due to the simultaneous saturation in quartz and two feldspars characteristic of the Bishop magma (see below), this time frame also includes the vast majority of feldspar crystallization, leading to the conclusion that the Bishop existed as a large-volume, crystal-poor magma body for a maximum of only a few thousand years.

### Thermodynamic and heat flow modeling

Different lines of evidence presented above suggest that quartz crystallization in the Bishop magma lasted only a few thousand years, and most of the crystallization occurred within the final 1,000 years before eruption. One significant question is whether such timescales are consistent with heat flow requirements, i.e. with the need to transport the heat of cooling and crystallization through the country-rocks.

#### Heat of cooling and crystallization constraints

It has long been argued that the Bishop magma is “eutectoid” in nature (e.g. [18]). In particular for early-erupted Bishop pumice, (1) the coexistence of quartz, sanidine, plagioclase, biotite, magnetite, ilmenite, and a H_2_O-CO_2_ fluid phase, and (2) the similarity in major-element composition between bulk pumice [6] and melt inclusions in quartz [18] strongly argue for nearly invariant behavior during crystallization.

**Figure 6:**
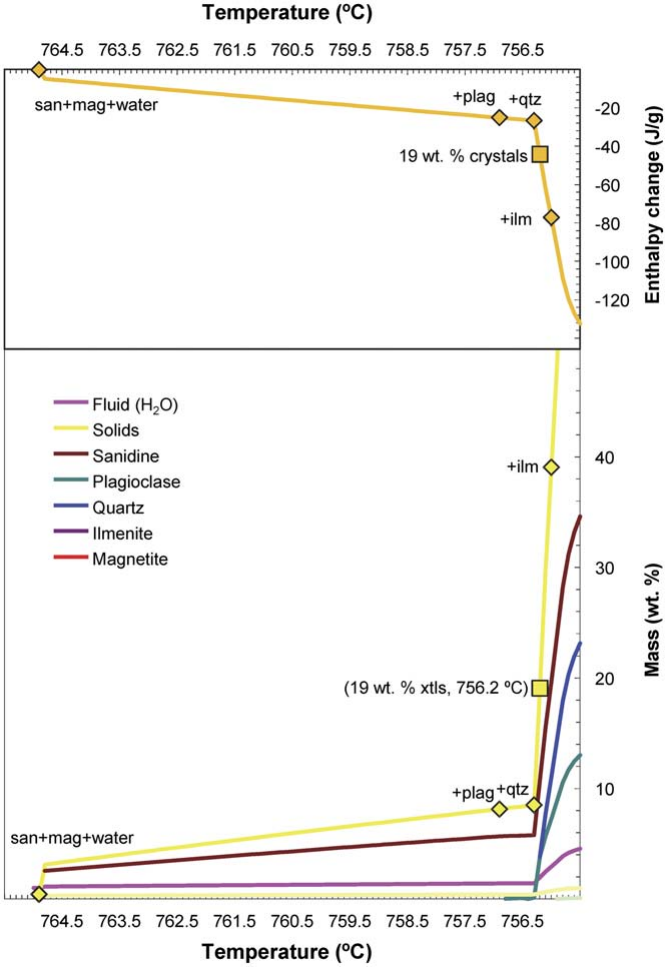
Thermodynamic and heat flow modeling results. Temperature (°C) versus enthalpy change (J/g; top panel) and versus abundance (wt. %; bottom panel) plot shows results of MELTS [22], [23] simulations. Initial composition is average late-erupted pumice composition from Hildreth [6]. Simulation assumes equilibrium crystallization at 175 MPa, under fluid-saturated conditions (see [23]), in agreement with melt inclusion data [18], [24]. Note short crystallization interval (<10°C). Nearly invariant condition is reached at 756.1°C when the system becomes saturated in quartz (in addition to fluid, sanidine, magnetite, and plagioclase), after which point crystallization is nearly isothermal. Quartz crystallization effectively locks the system at the nearly invariant temperature, given that, upon heating, temperature excursions above the nearly invariant temperature are only possible after complete resorption of quartz.

Using rhyolite-MELTS [23], a modified calibration of MELTS that better predicts the quartz+feldspar saturation surface as a function of pressure, we have simulated the crystallization of representative Bishop compositions under various conditions. In all cases, the expected nearly invariant behavior is well-captured by rhyolite-MELTS (see detailed discussion in [23]); once the system becomes multiply saturated with quartz, sanidine, plagioclase, and fluid, crystallization becomes essentially isothermal, with >50 wt. % crystallization over less than 1°C (Fig. 6). For early-erupted Bishop, the 0.5–12 wt. % crystals typically observed in pumice [6], [14], [50] require <1°C cooling, while late-erupted Bishop can achieve typical 12–25 wt. % crystals in <10°C (Fig. 6).

The importance of this nearly isothermal behavior is that the amount of heat that needs to be withdrawn is essentially limited to latent heat of crystallization, which rhyolite-MELTS calculates to be only 20–30 J/g (see Fig. 6). As a point of comparison, cooling these magmas by 50°C would produce an additional ∼65–70 J/g of heat, making the total amount of heat to be transported 3.5–4 times larger than the nearly isothermal case. Consequently, the nature of the heat-flow problem is significantly influenced by the invariant aspect of crystallization. Furthermore, nearly invariant crystallization effectively locks the system at a fixed temperature, given that temperature excursions are only possible after the nearly invariant phase (quartz, in this case; see Fig. 6) is completely resorbed.

#### Heat flow problem

Our rhyolite-MELTS simulations not only constrain the total heat of cooling and crystallization required to attain the observed compositions and crystal contents, but also confirm our expectation of nearly invariant crystallization. To contrast the behavior of invariant and non-invariant magmas, we employ well-known analytical solutions [51], which, rather than describing the systems in great detail, capture the essence of the problems of interest.

**Figure 7:**
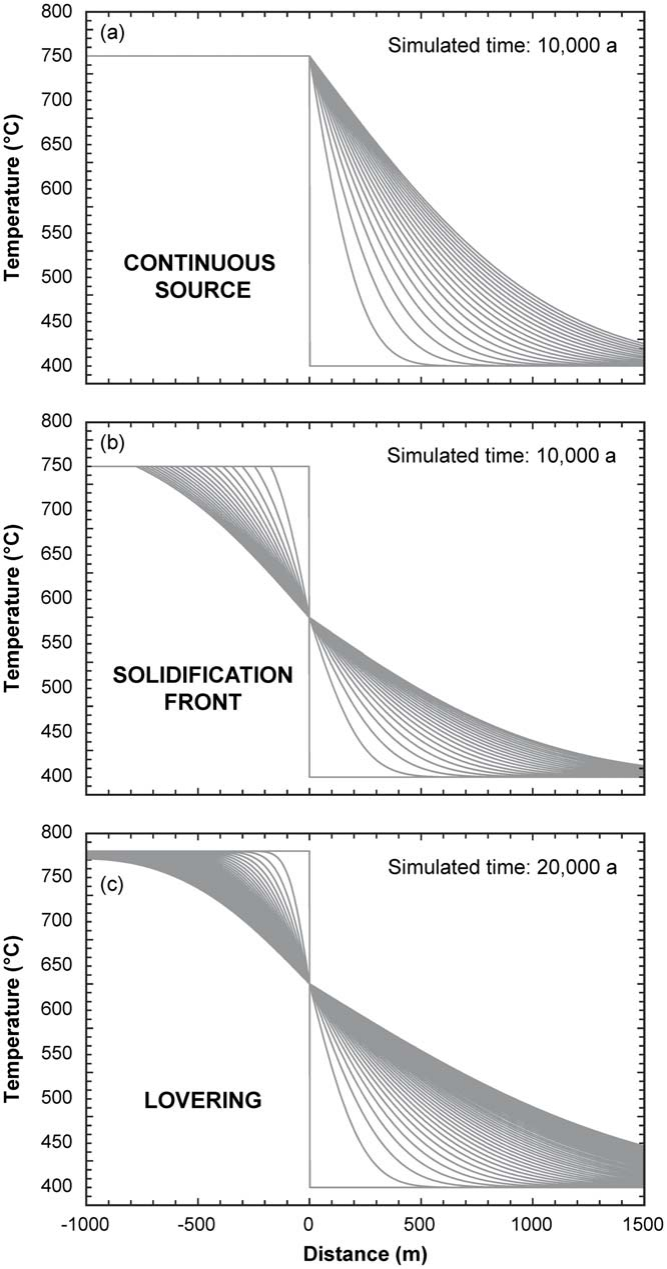
Results of heat-flow simulations showing the thermal evolution of a 1 km thick, semi-infinite magma column. Initial thermal profile is a step-function, with hot (750 or 780°C) magma on the left side and cool (400°C) country-rock on the right side. We use three different analytical solutions: (a) Continuous source, in which crystallization is dispersed throughout the invariant magma, and the magma—country-rock interface is maintained at its original position; changes in the thermal properties of the magma as a function of crystallization are neglected. (b) Solidification front, in which crystallization of invariant magma takes place from the magma—country-rock-interface inward; as a limiting case, thermal properties of the solidified zone are taken to be the same as those of the liquid. (c) Lovering [53], in which the specific heat of the melt (c_1_) is adjusted so as to simulate crystallization taking place linearly over the course of 50°C cooling; no correction is made to account for changes in thermal properties of initially molten zone as a function of crystallization. Simulation times as indicated; thermal profiles are shown every 500 years.

The Bishop magma body can be reasonably approximated as a 2 km thick body [18], [50] losing heat from both top and bottom. Importantly, the evidence accumulated to date for the Bishop Tuff suggests that renewed injections of mafic or felsic magma – common in many systems (e.g. [52]) – did not play a major role during crystallization of the Bishop magma body; no direct evidence has been found to date of co-erupted mafic material (e.g. [50]), and felsic magma additions during crystallization are inconsistent with the observed homogeneity of phenocrysts [6], except for late additions [50] possibly connected with the transition towards eruption [31]. Accordingly, we consider a 1 km thick magma column emplaced instantaneously in country-rock at 400°C, with heat loss solely through the top of the column (or, equivalently, a 2 km thick column with heat loss from top and bottom). We explore 3 different solutions (parameters used listed in Table 3):

1. *Continuous Source* (Section 2.4 of [51]). In this case (Fig. 7a), an invariant magma (pure substance or eutectic) is emplaced at the invariant temperature (assumed to be 750°C). Crystallization is dispersed through the melt and the temperature of the magma is kept constant. Heat transport through the magma is fast compared to heat transport through the country-rock. No correction is made for changes in the thermal properties of the magma due to gradual crystallization, but the effect is minimal given the small crystallization interval considered here. Progress of crystallization can be calculated from the heat flux at the boundary, given that all heat lost is latent heat.
2. *Solidification Front* (Section 11.2.IV of [51]). This case (Fig. 7b) is similar to the previous one, except that crystallization takes place along the melt-rock contact. Initially, melt is in direct contact with country-rock, but melt solidifies completely at the contact, and the melt-rock contact migrates inward as crystallization proceeds. While the new solid can have distinct thermal properties, we assume the limiting case in which the thermal properties of the new solid are the same as in the original melt. Progress of crystallization is calculated from the rate of migration of the melt-rock boundary.
3. *Lovering-type* [53]. In this case (Fig. 7c), magma is non-invariant. The effect of latent heat is accounted for by adjusting the specific heat so as to cause latent heat to be released gradually with cooling (see Table 3). In effect, this case is the antithesis of invariant crystallization, with crystallization proceeding linearly with temperature.

**Table 3:** Parameters used in heat-flow simulations.

Property	Country rock	Liquid
*(Units)*	*(CGS, °C)*	*(CGS, °C)*
ρ	2.6^a^	2.1^c^
c	0.21^a^	0.32^c^
K	0.006^b^	0.0036^b^
κ = K/(ρ*c)	0.011	0.0058
L	–	35^c^
c^*^ = L/50+c	–	1.02^d^

a Carslaw & Jaeger [51].

b Whittington et al. [74].

c Rhyolite-MELTS simulations.

d Only for Lovering-type simulation.

Interestingly, solutions (1) and (2) are two end-members of invariant crystallization. In (1), crystallization is dispersed within the melt, with no gradient in crystallinity within the magma, while in (2), melt is always crystal-free and crystallization takes place along the walls. Crystallization of an invariant magma is likely to be intermediate in character between these two models.

#### Heat flow and crystallization timescales

**Figure 8:**
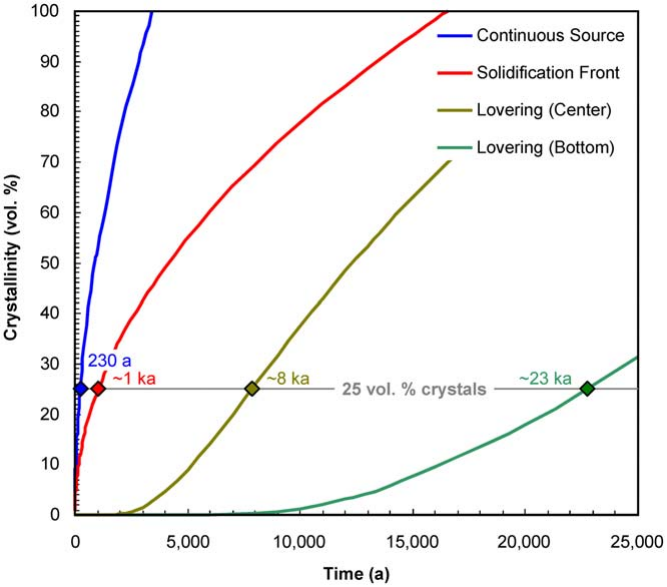
Evolution of crystallinity with time for the three solutions presented in Fig. 7 and discussed in the text. Notice the dramatic differences in behavior between the solutions for invariant magmas (Continuous Source and Solidification Front) and for non-invariant magmas (Lovering). Curves for Lovering-type crystallization are for the center and the bottom of the 1 km column. In particular, notice that significant crystallization (e.g. 25 vol. %) is attained in <1 ka for invariant magmas, in accordance with geospeedometry estimates presented in the text. Much longer timescales are required to cause significant crystallization of the interior of non-invariant magma bodies.

Application of the analytical solutions discussed above (Fig. 7) shows that both kinds of invariant crystallization lead to rather quick crystallization, with 25 vol. % crystals being attained within the first 1,000 years of evolution (Fig. 8). In contrast, in the non-invariant case, crystallinity is a function of temperature, and a continuum of crystallinity develops within the magma. Extended periods of time (>10 ka) are needed to start cooling the bottom of the column, by which point ∼1/3 of the column is fully crystallized, less than half has <25 vol. % crystals, and the bottom of the column is essentially crystal-free. Even for a point lying at half distance from the initial melt—country-rock contact, crystallization rates are much slower than for the invariant case, requiring ca. 8,000 years to attain 25 vol. % crystals (Fig. 8). Not only is non-invariant behavior inconsistent with our rhyolite-MELTS simulations, but the evidence for a continuum in crystal contents is entirely lacking in the Bishop Tuff. And, the relatively narrow range of crystal contents [14], [50] and the compositional homogeneity of phenocrysts [6] observed are quite consistent with nearly invariant crystallization.

The contrast in timescales between invariant and non-invariant magmas can be understood based on the differences between the two kinds of heat flow problems. For invariant magmas, all heat loss promotes crystallization, given that there is no sensible heat generation within the magma. Further, with magma temperatures buffered at the invariant temperature, thermal gradients at the magma-rock contact are steeper in the invariant case than the thermal gradients within the magma in the non-invariant case. It results that crystallization proceeds a lot more quickly in the invariant case. That invariant magmas can crystallize as much as 25 vol. % in ca. 1,000 years lends significant support to the timescales of crystallization estimated based on quartz geospeedometry.

## Discussion

### Implications for the longevity of giant magma bodies

Geospeedometry results suggest quartz crystallization on millennial timescales, and heat flow considerations suggest that these are the expected timescales for the problem of interest. These timescales contrast drastically with radiometric results (e.g. [10]) and with timescales derived from modeling of basalt-to-rhyolite crystallization trajectories using MELTS [54].

Ion probe U-Pb dating suggest zircon crystallization spanned over >100,000 years [10]. In contrast, TIMS U-Pb zircon crystallization ages combined with Ar-Ar eruption ages suggest that zircon crystallization within the final millennia of the Bishop magma history was substantial [11]; these results are contentious given the difficulties inherent in comparisons of U-Pb and Ar-Ar ages [12], and their potential significance has not been explored. The contrast between these zircon timescales suggests that they are in fact recording different facets of the evolution of the Bishop magma. Much of the success in using zircon as a geochronometer stems from its ability to survive through time, but we suspect that it is this unparalleled survival capacity that makes it a poor recorder of the lifetimes of large pools of crystal-poor magma. In order to generate a giant body of crystal-poor magma like the Bishop magma body, it is necessary to partially melt large amounts of crust [55] or to accumulate and crystallize large amounts of parental magma [56], and to segregate this melt from crystal-rich mush, which almost certainly leads to a very dynamic environment characterized by waxing and waning of the melt pool through time [55], [57], [58]. In such a scenario, it is not surprising that zircon will record a much more protracted history, as slow dissolution rates may limit resorption [59]. Further, zircon extraction from mush may be facilitated by its small size, leading to complex zircon populations [60]. It is thus much more likely that zircon in fact records the construction of these giant bodies of magma (see [61], [62] and references therein), which seems to take place over tens to hundreds of thousands of years, in agreement with recent studies of zircon ages in younger volcanic deposits [63], [64]. Quartz, on the other hand, records the crystallization of these large masses of segregated evolved melt in hundreds to thousands of years from a virtually crystal-free state.

Crystallization timescales on the order of millions of years have been recently suggested based on MELTS calculations and simple heat balance considerations [54]. Fowler & Spera [54] consider the derivation of high-silica rhyolite from basaltic parental magmas, despite the complete lack of evidence of such derivation, or even for interaction between mafic and felsic magma in the Bishop Tuff. They compute evolution over >400°C, with resulting large changes in melt and mineral compositions, none of which are recorded in Bishop Tuff minerals [6], melt inclusions [18], [25], or eruptive products [50]. The significance of their results, in the absence of physical evidence, is thus questionable. On a broad sense, if high-silica rhyolites derive from mafic magmas through step-wise fractionation (e.g. [56]), it is possible that the analysis by Fowler & Spera [54] provides information on the timescales required for fractionation, and it is intriguing that these timescales could be as short as ∼130 ka [65], similar to the timescales recorded by zircon. If, on the other hand, high-silica rhyolites are to a significant extent products of crustal recycling (e.g. [55]), then the timescales derived from the exercise conducted by Fowler & Spera [54] are meaningless. In any case, we argue that their analysis does not yield information on the longevity of a large body of crystal-poor rhyolitic magma, which our data strongly suggest to have evolved on millennial timescales.

Our interpretation has fundamental implications. The Bishop magma is characteristically zoned in many respects [6], with this zonation most likely reflecting crystal fractionation processes [66], [67]. The homogeneity of phenocryst compositions in individual pumice clasts implies that the magma body zonation predates crystallization [6], [50] and was established during emplacement or segregation of magma from a crystal mush. Evidence to date suggests that the Bishop magma was oversaturated in volatiles and contained >15 vol. % pre-eruptive bubbles in its uppermost regions [24]. In this context, the difficulty is not how to trigger a supereruption as much as it is how to prevent one from happening, especially with the timescales inferred from zircon geochronology. With quartz crystallization, bubble exsolution would also occur, with the potential consequence that the system could quickly evolve towards an eruptible state, preventing itself from residing in the crust for extended periods of time. The relatively long repose times – frequently hundreds of thousands of years [3] – observed in many volcanic centers are often taken as an indication of the longevity of giant magma bodies. Short repose times between supereruptions in some areas [68]–[70] provide circumstantial evidence that giant magma bodies can evolve quickly. In this sense, while zircon geochronometry indicates that it may take hundreds of thousands of years for the crust to be capable of accumulating and segregating large amounts of crystal-poor magma, our data suggest that the giant pools of eruptible magma thus formed are rather ephemeral features, which quickly and effectively destroy themselves during supereruptions.

### Conclusions

We use four lines of evidence to infer the timescales of crystallization of the Bishop Tuff magma body:

1. Timescales of relaxation of Ti profiles in quartz suggest quartz residence times of ∼500–3,000 years; crystal size distributions suggest that >99 wt. % of crystals present would have crystallized within this timeframe;
2. The coexistence of round and partly faceted (negative crystal shape) melt inclusions in quartz suggest that quartz residence times are similar to the timescale for melt inclusion faceting; calculated faceting times suggest quartz residence times of ∼500–1,500 years;
3. Using growth rates constrained by Ti relaxation times and known crystal sizes, we calculate crystallization times based on crystal size distributions of ∼500–2,500 years;
4. Thermodynamic considerations suggest crystallization of nearly invariant magma under essentially isothermal conditions; crystallization of such magma would be particularly efficient due to the absence of sensible heat contributions and steep thermal gradients, resulting in crystallization times of <1,000 years.

The agreement between these various estimates strongly supports crystallization of a giant magma body from a nearly crystal-free initial state over millennial timescales. We thus argue that giant magma bodies are ephemeral.

## Supporting Information

Movie S1 Animation showing 3D view of crystals in pumice chip (sample AB-6203F). Euhedral quartz and feldspar grains shown in green, magnetite in blue, and pyroxene±biotite in white. Note the overall trend of decreasing numbers of crystals with increasing size. Sample is approximately cylindrical, field of view ∼9 mm in diameter.
(MPG)
